# Gait Alteration in Individual with Limb Loss: The Role of Inertial Sensors

**DOI:** 10.3390/s23041880

**Published:** 2023-02-07

**Authors:** Andrea Demeco, Antonio Frizziero, Christian Nuresi, Giovanni Buccino, Francesco Pisani, Chiara Martini, Ruben Foresti, Cosimo Costantino

**Affiliations:** 1 Department of Medicine and Surgery, University of Parma, 43126 Parma, Italy; 2 Division of Neuroscience, IRCCS San Raffaele, University Vita-Salute San Raffaele, 20132 Milan, Italy; 3 Department of Human Neuroscience, University la Sapienza Rome, 00185 Rome, Italy; 4 Department of Diagnostic, Parma University Hospital, 43126 Parma, Italy

**Keywords:** amputation, inertial measurement unit, rehabilitation

## Abstract

Amputation has a big impact on the functioning of patients, with negative effects on locomotion and dexterity. In this context, inertial measurement units represent a useful tool in clinical practice for motion analysis, and in the development of personalized aids to improve a patient’s function. To date, there is still a gap of knowledge in the scientific literature on the application of inertial sensors in amputee patients. Thus, the aim of this narrative review was to collect the current knowledge on this topic and stimulate the publication of further research. Pubmed, Embase, Scopus, and Cochrane Library publications were screened until November 2022 to identify eligible studies. Out of 444 results, we selected 26 articles focused on movement analysis, risk of falls, energy expenditure, and the development of sensor-integrated prostheses. The results showed that the use of inertial sensors has the potential to improve the quality of life of patients with prostheses, increasing patient safety through the detection of gait alteration; enhancing the socio-occupational reintegration through the development of highly technologic and personalized prosthesis; and by monitoring the patients during daily life to plan a tailored rehabilitation program.

## 1. Introduction

Limb amputation has a high impact on a person’s quality of life, affecting the patient’s independence in activities of daily life, mobility, and sociality [1]. It is estimated that 1.6 million patients live with the loss of a limb, and the rate is continually growing [2]. Limb amputations can be divided in two grades: major limb loss, involving long bones, such as the humerus, radius, femur or tibia, and minor limb loss, involving at various levels, the hand or foot. Vascular diseases, trauma, cancer, and congenital abnormalities are the principal causes of a limb amputation [2]. Although studies on the trend of limb amputations are sometimes contradictory, amputation remains an issue concerning both patient care and healthcare costs [3,4], considering that the average hospitalization after amputation is about 21 days, and rehabilitation can take up to six months for the final prosthesis [5]. Patient satisfaction, functional utility, and aesthetics are the principal goals in prosthetic restoration [6]. A mean wearing time of 9.3 ± 5.5 h per day is estimated for patients with prostheses, thus the correct fitting, function, and education of the patient are fundamental to increase compliance and functional mobility [7]. On the other hand, many patients stop using prostheses after the rehabilitation course [6,8] due to the occurrence of residual limb complications including overuse syndrome, degenerative conditions, and injuries [9].

In particular, lower limb amputations are known to affect gait and balance in patients usually presenting several comorbidities, e.g., diabetic neuropathy, ulcers, and older age that further increase the risk of falling [9]. Upper limb amputation accounts for approximately 8% of all limb amputations. They are less common than lower limb amputation since they are frequently related to traumatic events and soft tissue tumors. However, they have a huge impact on the quality of life since they reduce the ability to interact with the environment, and perform specific work tasks [10].

A correct prosthesis application and a structured rehabilitation program are essential to achieve the maximum level of patient functionality [11]. The main focus is to improve walking in a patient with a lower limb prosthesis or to favor the prosthesis control accuracy [12]. The rehabilitation program includes strengthening exercises, gait training, virtual reality, balance training, and proprioceptive exercises [11]. However, the starting point to plan a tailored program is represented by the functional evaluation of the patient through methodological and validated outcome measurement scales, e.g., Trinity Amputation and Prosthesis Experience Scales (TAPES), ICF, or functional tests, e.g., 6MWT for fall risk assessment [6,13,14]. In this context, scientific progress has enabled the development of accessible, small-scale technologies capable of recording body movement in a more sensible and objective way compared to the ordinal scores of “semi-quantitative” clinical scales [15]. These tools have been successfully utilized in rehabilitation medicine to assess different aspects of a patient’s daily life and to measure therapeutic interventions. The first application to measure human motions is linked to Saunders, in 1953, with the aim of describing normal and pathological gait [16]. The gold standard of motion analysis is represented by the optoelectronic system with an infrared camera to capture motion. Often integrated with surface electromyography and force platforms, they require specially dedicated spaces, and are usually inapplicable in clinical practice [17].

Moreover, in recent years, emerging technologies have been utilized in movement analysis, with new and less expensive systems based on RGB and/or RGB-D cameras (e.g., Microsoft Kinect V2, Microsoft Azure), able to provide a detailed gait and posture analysis through a markerless approach that facilitates their use in clinical practice [18,19,20]. However, these systems still present some limitations, e.g., most of them are only validated on healthy subjects, there is the need of a particular framing of the subject, and only short walking distances are taken into account [20].

Conversely, Inertial Measurement Units (IMU) are wearable, and a recent systematic review and meta-analysis demonstrated a good correlation of many kinematic parameters between IMU and the gold standard [21], and are able to provide information on gait or limb kinematics, the measurement of joint movement angles [22,23], and the evaluation of performance in athletes [24,25].

Inertial sensors play a key role especially in the development of new assistive technologies [26,27,28], they are classified into two major families: (i) accelerometers that measure linear acceleration; and (ii) gyroscopes that measure angular velocity. Most accelerometers and gyroscopes are designed using MEMS (micro-electro-mechanical systems) technology, which allows a reduction in sensor size and thus a wider range of applications [29,30]. Often these two triaxial sensors are combined into a single measurement unit (six-degree-of-freedom IMU or 6-DOF IMU) integrating the two types of information. In some cases, they can be supported by the presence of a magnetic field measurement system that allows the assessment of body orientation in space (9-DOF IMU or MIMU) [31].

To date, notably in the last 10 years, IMU has been utilized in the evaluation of amputee patients and the development of technologically advanced prostheses with the aim of analyzing the patient’s deficits and providing a customized solution, reducing the whole range of complications related to both amputation, and stump prosthetics; however, their applications have yet to be clearly delineated. Considering the positive effect that these smart technologies could have in modifying the overall quality of life of both upper and lower limb in amputee populations, the aim of this narrative review was to condense the current state of the art regarding the possible applications of IMU in the amputee patient population.

## 2. Materials and Methods

Two authors examined the following databases: Pubmed, Embase, Scopus, Google Scholar, and Cochrane Library. The selection of articles was conducted throughout the search string: “amputee”; “prosthesis”; “artificial limb” [MeSH]; “amputation” [MeSH]; IMU, “inertial sensors”; “accelerometer”, “Monitoring”, “Physiologic”, “instrumentation” [MeSH], and the Boolean operators AND and OR. We considered only articles published from 1 January 2010 to 31 November 2022 with the full text. The results of the search yielded 580 results. The selection was determined considering: 1. Patients with mono/bilateral limb amputation in the upper and/or lower limbs; 2. The use of inertial sensors; and 3. Interventions that could have implications from the clinical point of view and/or quality of life of the patient. Disagreement in the study selections between the investigators was solved by a third author.

### 2.1. Data Extraction

Cochrane Review Group guidelines were utilized to conduct data extraction with an Excel document to evaluate inclusion criteria. Full texts were examined, and records were collected in the document. Authors; publication year; participant characteristics; design of studies; outcomes; main results, were extracted from the included papers.

### 2.2. Methodological Assessment

We utilized a modified version of the STROBE criteria to conduct the methodological assessment, through ten criteria. Disagreements were assessed and solved by consensus. A numerical categorization (1 if present; 0 if non-present) was utilized for item assessment. The studies were classified with a high risk of bias with a score of <7 and considered at low risk of bias with a score of >7. We considered previous guidelines, the research questions, appropriate evidence, the studies’ qualities, results synthesis, and their correct interpretation to conduct the clinical review [32,33].

## 3. Results

### 3.1. Study Selection

**Figure 1:**
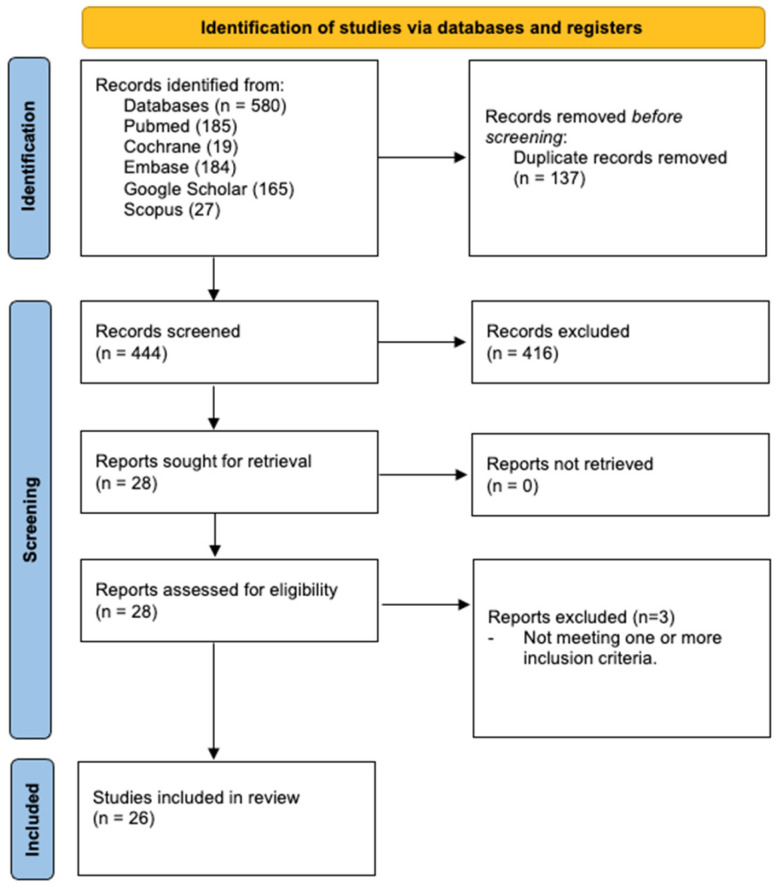
Prisma Flow Diagram.

A total of 580 records were identified after the database search, as indicated in the PRISMA flow diagram in (Figure 1). After duplication deletion, and the assessment of 444 studies, 26 were considered to be eligible, divided into four main topics: (1) the analysis of movement, (2) the risk of falls, (3) the study of energy expenditure, (4) the development of sensor-integrated prostheses.

### 3.2. Main Characteristics of the Studies

Table 1 summarizes the main characteristics of the studies. Twelve out of 26 studies [34,35,36,37,38,39,40,41,42,43,44,45] investigated different spatial and temporal parameters of Gait. Specifically, one study [34] focused on the evolution of kinematic parameters during a 6MWT and found that the prosthetic limb has greater toe clearance and that this is also more variable. A study [35] described the use of a new algorithm to be implemented when using inertial sensors to record gait events in real time with 100% accuracy in recording initial contact and toe off. On this topic, Wentink et al. [37] also demonstrated how the use of IMUs is reliable in recording the onset of walking in both the healthy limb and the amputated limb [36]. The study investigated the use of IMUs in joint angle calculation, demonstrating good correlation of measurements with 3D motion detection systems. Two studies [38,39] focused on the recording of accelerations: Simonetti et al. [38], using a network of five MIMUs, derived individual SCoMs calculating the BCoM with a moderate to strong correlation with force platforms; Paradisi et al. [39], used three MIMUs to detect acceleration in three different body segments (head, thorax, pelvis) demonstrating an alteration of the normal head-to-pelvis accelerometric gradient in LLP patients and deriving an attenuation coefficient that could be useful for assessing the mobility of amputee patients. One study [40], evaluated flat and slope walking speeds through a single IMU integrated in an MKAP with a good RMSE in both walking situations.

Ref. [41] investigated the use of accelerometers in the measurement of step length with no conclusive results in validation with force platforms, while demonstrating a good correlation with referenced literature measurements. Ref. [42] demonstrated how the use of accelerometers can differentiate between different states of dynamic stability (e.g., while walking on level ground or on uneven ground) and correlated the various differences in accelerometer data with clinical outcomes. As shown by Lamoth CJ et al. [43], trunk acceleration and walking speed are the main factors influencing gait stability and variability. Finally, regarding symmetry and regularity, Tura et al. [44] showed that step and stride duration as well as step and stride regularity are good indices of walking regularity and symmetry; in conclusion, Clemens S. et al. [45] used various data on lower limb accelerations to derive two indices (SSS and SRS) that are also useful for assessing gait symmetry and regularity.

Three out of twenty-six studies [14,47,48] used inertial sensors to classify and assess the risk of falls. Daines KJF et al. [14] used a random forest classifier and a smartphone to classify the risk of falls in amputees with interesting results in terms of sensitivity and specificity. Shawen et al. [47] used a machine learning approach with fewer false alarms than a threshold algorithm approach. In addition, Hordacre et al. [48] used a Stepwatch 3 activity monitor coupled with a GPS to monitor non-fallers and amputee fallers to demonstrate a reduced participation in various social contexts.

Six out of twenty-six studies [49,50,51,52] investigated the implementation of IMUs in advanced prostheses to improve their control and fluidity during daily tasks. Four studies focused on implementing data recorded from IMUs for LLP. Akin, O.K. et al. [49] studied the movement of healthy limbs using two acceleration sensors and implemented data collected in an algorithm useful for controlling the trajectory of a mechanical prosthesis. Chang et al. [50] collected data from an IMU embedded to a prosthesis with mechanical ankle control and a load cell recording ground reaction forces, for terrain detection using fuzzy logic systems. Ref. [51] proposed a system made of three IMUs for detecting intent recognition using a convolutional neural network system. Finally, Ref. [52], using an IMU and a vibrational feedback system, stimulated an illusion of movement in the prosthetic limb. Two studies focused on the use of IMU to improve the use of upper limb prostheses. Krasoulis et al. [53] studied a multigrip classification system through two sEMG-IMUs sensors in healthy and TRA participants with a potential application in grip control for upper limb prostheses. Sharba et al. [54] proposed a shoulder movement classification system through IMUs and sEMG placed on a shoulder girdle with the aim of helping patients with more proximal amputation controling prosthesis.

Finally, five studies out of twenty-six [55,56,57,58,59] investigated the potential role of IMUs in deriving energy expenditure. Ladlow et al. in two studies [55,56] validated a system composed by activity monitor and HR monitor for assessing energy expenditure in LLA patients by comparing data with indirect calorimetry [55], especially if the activity monitor is placed on the side of the pelvis above the amputated limb hip [56]. Refs. [57,58] studied step count on the LLA population. Smith JD et al. confronted different types of sensors and activity monitor finding that Stepwatch positioned on the ankle and Vivofit on the wrist provided the most accurate step count. Desveaux et al. [58] demonstrated that LLA patients, despite a good recovery in mobility, walk less than 6500 steps and tend to avoid vigorous physical activities. Lastly, Kim et al. [59] found no differences in energy expenditure between powered prosthesis and non-powered prosthesis patients, although a reported faster pace with powered prosthesis was observed.

### 3.3. Methodological Quality

Table 2 reports the methodological quality scores of the studies [29]. The overall quality of the studies considered was high. Seven studies [34,37,48,55,56,57,59] had 10 points; height studies [14,38,39,40,41,45,47,58] had nine points; one study had eight points [52]; seven studies [35,42,43,44,51,53,54] had seven points; one study [49] had six points; and two studies [36,50] had five points.

## 4. Discussion

### 4.1. Gait Analysis

The importance of walking is particularly evident during daily activities, and highly influence a patient’s independence and participation [60,61]. Amputation, and consequently lower limb prosthesis (LLP), profoundly modifies the gait pattern [62]. Furthermore, the establishment of compensatory movements, in the long term, could generate low back pain and negatively affect the patient’s quality of life [63]. The main discrepancies in the gait of patients with lower-limb amputations depend either on malalignment or length differences with the result of modifying the kinematics of walking and increasing its metabolic cost [62]. Aspects such as the kinematics of the body’s center of mass and its acceleration in space have crucial importance for the patient’s rehabilitation. The use of portable sensors has increasingly attracted the curiosity of the scientific community to evaluate the kinematics of the gait [64], allowing the physician to obtain a detailed assessment of the patient at the beginning, during and at the end of their rehabilitation program, taking into account various parameters including linear and angular acceleration, walking speed, step cadence, and gait events [34,35].

#### 4.1.1. Joint Angle Calculation

Inertial sensors have been successfully studied to assess joint angles in both healthy and amputee patients. In their study, Seel T. et al. [36] used inertial sensors to infer the joint angles of a transfemoral amputee patient wearing an articulated knee prosthesis and compared the results with optoelectronic detection systems. The authors found high sensitivity in this measurement, demonstrating that the IMU could potentially be used on both the healthy and prosthetic limb; however, the small sample considered could affect the generalizability of the conclusions [36].

#### 4.1.2. Detection of the Onset of Gait

Wentinik et al. [37], by combining data recorded using IMU, insole sensors and surface EMG (sEMG), assessed the onset of walking in both healthy and prosthetic limbs. The use of IMUs integrated with sEMG would seem to predict the onset of walking in these patients, both when the healthy limb and the prosthetic limb advance. In the case of the prosthetic limb advancing first, the data collected by the accelerometer module were found to be more accurate, whereas in the case of the healthy limb data collected by the gyroscope module were more reliable [37]; in addition, sEMG has shown to anticipate the initial movement prediction, providing an useful information for prostheses control. However, this was valid only when the prosthetic leg led.

The results of this study pave the way to the development of gait intention detection systems at the base of future neural-integrated and controlled prostheses [65].

#### 4.1.3. Acceleration and Walking Speed

Simonetti E et al. [38] in a recent case study compared MIMU with optometric gait evaluation systems and force platforms to validate their accuracy on one subject with transfemoral amputation. The authors applicated different MIMUs on the seven major segments (the trunk, thighs, legs, and feet) contributing to 3D Body Center of Mass (BCoM) acceleration in the transfemoral amputation patient. The patient were asked to walk with a gait speed of choice on a force plate and calculated the acceleration by integrating the individual Segment Center of Mass (SCoM) accelerations, concluding that the use of a network of five MIMUs shows promising results for measuring the three-dimensional acceleration of the BCoM and its velocity with a strong correlation to force platforms and optometric systems [38]. Patients with transtibial amputation and those with transfemoral amputation have similar gait patterns, characterized by a symmetrical increases in step length and width, double stance duration, pelvic obliquity, lateral trunk flexion, and anteflexion of trunk and pelvis. However, patients with transfemoral amputation present a reduction in stance phase and an increase in swing phase duration of the prosthetic limb. Furthermore, in these patients, there is an increase in ground reaction forces during the stance phase and a shift of the center of activity on the intact limb side. Despite the common features, the level of amputation influences the walking pattern of patient, representing a way to compensate the lack of ankle joint or knee joint [66]. These compensatory movements, always asymmetrical, are rarely localized in lower body segments, and usually involve higher districts of the body such as the trunk and head. In healthy patients, physiologically, lower body accelerations are transmitted in a decremental caudo-cranial gradient. In a recent study, Paradisi et al. have investigated how this gradient is modified in patients with inferior limb amputation placing IMU (1) at the level of the lambdoid suture of the occipital bone; (2) at the center of the sternum, and (3) at L4-L5 just above the pelvis of the participants. They found prosthesis-related functional asymmetry due to the inability to perform plantar-dorsiflexion movements with the prosthetic foot leading to exaggerated proximal joint kinematics (pronounced pelvic tilt, lumbar hyperlordosis) during stance. These modifications would then lead to a larger coefficient of attenuation of pelvic-sternal accelerations in the dynamic phase of stance. In particular, amputees have a higher mediolateral acceleration, which influences a reduced deceleration gradient in the substernal regions but is also responsible for a higher head acceleration [39]. In this study, the authors utilized sewn pockets to place IMU, that could introduce possible errors, and considered only indoor environments.

Walking speed represents a relevant indicator of the patients’ performance. Amputees have a reduced walking speed compared to non-amputees and a higher level of amputation is related with a lower walking speed that might represent a strategy to increase stability and reduce the medio-lateral acceleration that these subjects experience. Due to difficulty to record directly the walking speed in real life conditions, because of the difficulty to assess spatial parameters from wearable sensors signals, this parameter is usually estimated through the cadence, step counts and activity bout duration. Nevertheless, a recent article of Dauriac et al. proposed a new model to estimate the instantaneous walking speed of patients with transfemoral amputation obtaining an accuracy of 9% through an IMU placed on the tibia and body height [40]. However, the validation of this model should be completed in a real environment and during real-life activities.

#### 4.1.4. Step Length

Step length (SL) is also a useful parameter to evaluate gait in patients with lower limb amputation, providing a measure of the patient’s walking ability and evaluating the symmetry of the steps and the distance covered. Matthew J. Major et al. [41] tried to develop an SL measurement system based on accelerometers. In their study, an accelerometer was applied at the level of the fourth lumbar vertebra (BCoM) in healthy subjects and in subjects with lower-limb amputations (transtibial and transfemoral mono and bilaterally). In healthy subjects, the inertial measurement system was also considered with an optometric gait analysis system. Although inertial sensors have a good test-retest reliability in healthy patients, showed contrasting values in patients with LLP, in prosthetic patients, the walking distance was generally over- or under-estimated (with an error of sometimes up to 30%); however, the SL of prosthesis users coincided with the data present in the literature. This error was greater in those patients with walking compensation strategies such as circumduction or hip advancement and therefore did not fall within the sagittal range [41,67,68,69].

#### 4.1.5. Variability and Stability, Symmetries and Regularity

Amputation affects both motor and sensory functions, altering the lower limb complex feedback mechanisms regulating the whole gait cycle, including its variability, regularity and symmetry, and increasing the risk of falling due to the lack of dynamic stability [70,71]. Several approaches have been proposed in the literature, with different IMU localization, limiting the comparison of the results. To investigate the relationships between dynamic stability and acceleration, Howcroft, J. et al. [42] recorded acceleration with a single sensor positioned at pelvic level on two different types of terrain (level ground and uneven ground). The authors found statistically significant differences in temporal parameters (stride time and cadence), acceleration data (vertical acceleration range and maximum vertical acceleration), and the mean-lateral harmonic ratio between walking on level ground and uneven ground. Moreover, they identified three parameters, ML acceleration, AP acceleration standard deviation and stride time, that were highly correlated with balance and mobility clinical scores [42].

Similar results are described by Lamoth, C.J. et al. [43] in a study evaluating trunk accelerations and stride time, which are indicators, through their variation in time, of dynamic stability in walking. The study used inertial sensors recording antero-posterior and medio-lateral accelerations by fixing a tri-axial accelerometer at the level of the third lumbar vertebra. In this study, they found statistically significant differences between amputees and healthy patients in trunk accelerations and walking speed [43]. As also described in other works [72,73], amputees show significantly larger mid-lateral accelerations and, consequently, larger lateral oscillations, two parameters that represent a reliable method of analyzing the gait stability of LLP.

In addition, Tura et al. [44] demonstrated, using a single sensor fixed at the level of the xiphoid process, that step and stride length as well as step and stride duration, can be used as reliable indices of gait regularity and symmetry [44].

Moreover, in a more recent study, Clemens S. et al. [45] calculated the symmetry and the repeatability of the walk during the 10 Minutes Walking Test (10 MWT) through the use of two sensors placed both on the healthy limb and on the prosthetic amputee. The authors in this case, by recording angular velocities at the leg and thigh and using specific algorithms, were able to evaluate the between limbs differences in angular velocity and summarized them in a score of symmetry (segmental symmetry score—SSS) and repeatability (segmental repeatability score—SRS). These scores were found to have good test-retest reliability in detecting differences in movement between the prosthetic leg and intact leg (in terms of SRS, SSS and angular velocity difference repeatability, AVD-R) with a limitation in detecting the differences between transfemoral and transtibial amputees. Trans-tibial amputees presented with greater AVD-R and lesser SRS in the prosthetic leg side versus the intact leg side. Transfemoral amputee, moreover, presented no differences in SRS but greater thigh AVD-R in the prosthetic side versus the intact leg side [45].

### 4.2. Fall Risk Classification

According to the World Health Organization (WHO), falls are the second leading cause of accidental death [74]. Following the amputation of a lower limb, the risk of falls is increased [75,76].

However, during daily life, the risk is higher than in the post-surgical phase, estimated to be three times higher than the rest of the population. This could be influenced by the development of compensatory strategies that increase the energy consumption during walking, leading to a premature muscle fatigue and loss of stability. The most important risk factor is gait variability. In healthy adults there are small and physiological variations in gait parameters. To the contrary, in patients with diseases that profoundly alter the gait (i.e., Parkinson’s disease, Alzheimer disease, frailty and amputation), these fluctuations become more pronounced, putting the patient at risk of accidental falls [76,77]. It should highlighted that most falls occur within the walls of the home [78] and therefore, it is very important to evaluate patient not only in an outpatient or gym setting but also during daily life.

In this context, wearable sensors (including inertial sensors) have been used with the intent of classifying fall risk in the amputee population, with high heterogeneity of results, due to the necessity of experienced personnel in their management, the low compliance of the patient, and the lack of standardized protocols (most studies in this field use different technologies and algorithms), as shown by Subramaniam, S. et al. [79].

In a recent study, Kyle J.F. Daines et al. [14] proposed the use of a smartphone as an inertial sensor, since they usually include an inertial sensor and sometimes also a magnetometer. In their study, an android smartphone was placed at the level of the posterior medial portion of the pelvis, and through a dedicated custom application recorded gait during a six-minute walking test (6MWT), computed with various classification systems, among which the random forest classifier and a correlation-based selection on the turn step future showed high specificity but low sensitivity [14]. The use of highly available devices is interesting but it is still difficult to apply in daily life because of the low replicability of patient’s sensor positioning [80,81]. In this context, a machine learning approach could overcome this limitation by considering the positions commonly used during everyday life (hand, pocket or pouch) extending the observation to the patient home [47].

The flexibility of using accelerometers could lead to an advantage for the clinician, who, by assessing the patient in their daily life environment, could estimate the risk of falls and act accordingly (e.g., with balance training). IMUs have been used not only to classify the risk of falls but also to monitor their impact on everyday life. Hordacre, B. et al. [48] demonstrated, through the use of a GPS and accelerometer, that amputee patients who experienced falls reduce their life and community participation, preferring home-based activities [48].

### 4.3. Prosthesis Development

Another field of particular interest regarding IMU concerns their potential role on reproducing a physiological walking pattern in the development of active prostheses that use technology to support the patient’s walking by reproducing as closely as possible the kinematics of the contralateral segment [82,83].

Kapti et al. [49] devised an experimental transtibial prosthesis by combining a servomotor and a series of elastic elements to allow plantarflexion and dorsiflexion movements compatible with a physiological gait cycle, as well as the control of the trajectory of the ankle joint. They used an inertial sensor positioned at the level of the ankle joint in healthy participants to obtain a symmetrical movement of the prosthesis and the contralateral limb. On the other hand, it is limited to patients with a normal biomechanical behavior of the healthy limb, and the assumption of a symmetrical gait pattern, not valid in amputees [49]. In this context, the IMU could represent a proprioceptive afference signal, measuring joint angles and prosthesis information [49].

Chang, M. et al. [50], in their study, proposed the use of inertial sensors and algorithms based on fuzzy logic to design a transtibial prosthesis sensitive to variations in the terrain. Five different environments were used: flat, upslope, downslope, upstairs and downstairs. The combination of the smart prosthetic ankle system and the fuzzy logic algorithm resulted in an accuracy of 97.5% in recognizing and adapting the gait in different settings, only if the user carefully walked following the authors’ guidelines [50].

One of the most important features of an active prostheses is the smoothness in locomotion changes [84] achieved by several sensors and computational strategies, such as machine learning. Ben-Yue, Su. et al. [51] attempted to use a different method to record movement intention in subjects with transfemoral amputation by using IMU. Thirteen motion states were investigated, such as simple level walking, climbing, or descending stairs, and changing from climbing stairs to walking on level ground. The data, recorded in a standardized laboratory setting by the sensors, were finally integrated into a convolutional neural network (CNN), investigating both steady and transitional motion states with an average accuracy in recognizing movement intention of 94.15% and a maximum accuracy 97.19% [51].

Another interesting application of IMU in LLP is the integration of the position sense of the prosthetic limb by using vibratory feedback to trigger the sensation of movement. Known as kinesthetic illusion (KI). McNiel-Inyani, Keri et al. [52] in their study used a vibratory feedback system activated by the registration of movements on a single axis to generate a KI, that participated in the integrity of the tendons through the stimulation of neuromuscular spindles [52]. However, the sensor drift could result in false triggers, and the device utilized caused a significant delay in KI.

Moreover, IMUs can also represent an implementation of the information of myoelectric prostheses of the upper limb, consisting of a sensor registering the electrical activity of the external surface of the stump and a prosthetic hand with motorized fingers which allows a grip obtained and controlled by the activity of the residual muscles [53].

Krasoulis, A. et al. [53] in their study used a custom-made EMG system integrated with MIMU for recording the electrical activity of the stump, the acceleration, orientation, and angular velocity of the stump. Thanks to this combination of sensors, they were able to record and classify the grip control of hand prostheses in patients with trans-radial amputation. The number of completions of simple “pick and place” tasks, and the time, were used to determine the control performance of the prosthetic hand. In addition, data collected seemed to open new perspectives on the implementation of real-time grip control systems to minimize unwanted prosthesis activities that would lead to inaccuracies in task execution [53]. Moreover, for patients with less conservative amputations, and therefore lacking the whole arm muscles to control their prosthetic hands, Sharba, G.K. et al. [54] studied a real-time classification system of five classes of shoulder girdle movement using a system of electromyographic electrodes and accelerometric recordings. Five movements were selected: (1) elevation; (2) depression; (3) protraction; (4) retraction; and (5) rest. The movements were recognized in real time with an accuracy of 92.8%, to allow a myoelectric control of a prosthetic hand in this kind of amputation [54].

### 4.4. Energy Expenditure and Metabolism

As mentioned above, amputation leads to increased walking energy expenditure. Transtibial prosthetic patients have significantly higher (20%) oxygen expenditure than healthy subjects, and the cost of oxygen consumption increases in proportion to the height of the amputation (maximum consumption for hemipelvectomies), as gait becomes progressively less efficient. In the last decade, therefore, research on prostheses has also focused on the development of technologies to reduce energy costs and oxygen consumption in patients with various levels of amputation [85].

The “gold standard” method for determining energy cost is indirect calorimetry. However, this method is expensive and impractical for use outside of hospital or clinical settings [86]. In this context, technology advancement has led to the development of new multimodal sensors that record both classic inertial data and heartbeat, whih were successfully utilized to monitor the physical activity of healthy subjects [87]. However, there is only one study in the literature published by Ladlow, P. et al. [55] validating this approach on amputee patients, integrating two types of sensors (ActiHeart and GT3X+), that showed a good correlation with indirect calorimetry [55,56].

Recently, Smith, J.D. et al. [57] utilized a series of integrated sensors (ActiGraph™ GT9X Link; Garmin Vivofit; Modus Stepwatch) placed on the lateral part of the residual limb, assessing, through the 2-m walking test (2MWT), the step count and oxygen consumption in patients with single and bilateral transtibial or transfemoral amputation. They found no differences between tibial and femoral amputees in heart rate, VO2 and perceived exertion, but there was a statistically significant difference in step count, higher in transtibial amputees [57], and a higher oxygen demand in patients with transfemoral amputation [88]. In healthy subjects, the simplest parameter correlating with physical activity is the daily step count, accounting for 10,000 steps positively correlated with a reduction in mortality and cardiovascular disorders. For subjects with chronic diseases, this step count is lower (about 6.500). In particular, lower limb amputees perform much less physical activity, as evidenced by the study of Desveaux, L. et al. [58], in which they were estimated, through inertial sensors (Stepwatch monitor) and clinical measures, to have an alarming step count below 6500 [58].

Moreover, Kim, J et al. [59], addressed the question of whether powered prostheses could reduce the energy cost of walking in amputees during walking, but they found no differences in metabolic cost, total number of daily steps, walking speed, and perceived mobility between patients using powered prostheses and patients wearing non-powered prostheses [59].

## 5. Conclusions

Taken together, the findings of the present narrative review showed that IMU might play a key role in monitoring amputee patients. In particular, the detection of gait alteration could guide the physician during the rehabilitation plan and reduce the risk of falls. However, most of the studies considered are based on limited sample sizes, utilizing laboratory conditions and healthy participants. Moreover, there is a high heterogenicity regarding the type and localization of the IMUs, the algorithm utilized for gait analysis and movement prediction. On the other hand, the advancement in prosthesis design and the development of new sensors for myoelectric prostheses could have a positive impact on the functioning of patients. Further studies are needed to bridge the gap between technologies and clinical advancement by improving the crosstalk between engineering and medicine.

## Figures and Tables

**Table 1:** Main characteristics of the included studies. LLA (lower limb amputees); ULLA (unilateral lower limb amputees); BiLLA (bilateral lower limb amputees) (TFA (transfemoral amputees); TT (transtibial); TTA (transtibial amputees); BiTTA (bilateralt transtibial amputatees) TKA (through the knee amputees); LLP (lower-limb prosthesis); TAA (total ankle amputees); TRA (transradial amputees); HlULA (High-level upper limb amputee).

Article	Study Design	Participants	Aim	Procedure	Outcome
Beausoleil S. et al., 2019 [34]	Case Study	15 LLA Patients	To assess kinematic gait parameters during 6MWT and clinical applicability of IMU.	Post-Rehab assessment of gait, during 6MWT, with IMU (3D Acc + 3D Gyro) on both feet.	High stance and cadence variability on both limbs. High and variable minimal Toe clearance on AL. Gait kinematic parameters variability are correlated with future falls. Relevant IMU applicability in clinical context.
Maqbool H.F. et al., 2017 [35]	Controlled Clinical Trial	8 Healthy Control, 1 TFA Patients, 1 TTA Patient	To evaluate the reliability of RT Gait event detection algorithm in both flat and inclined surfaces for TFA patients.	Patients walked for 10 min at self-selected walking speed over flat surfaces and walk up and down a ramp (5° inclination) with a 6-DOF IMU (Acc + Gyro) fixed on the shank and insole with footswitches. The algorithm was written in MAT-lab.	100% detection accuracy for Initial Contact and Toe Off with different prostheses. Reliable algorithm for gait event detection.
Seel T. et al., 2014 [36]	Case Study	1 TFA Patients	To asses Joint Angle using IMUs and validate it’s measure with a 3D optoelectronic movement detection system (MDS).	IMUs (Xsense MTw) mounted on proximal and distal legs as well as foots. Body marker for 3D MDS (Vicon V612) mounted on both legs. Participant is requested to walk 10 m at self-selected speed. Data are gathered and confronted with 3D MDS	Joint angle calculation using accelerometer and gyroscope showed high precision and correlation with 3D MDS (1° RMSE for prosthetic leg and 3° for contralateral).
Wentinik E.C. et al., 2014 [37]	Case Study	3 TFA, 3 TKA	To detect onset of gait with IMUs.	Footswitches positioned in the heel center and under the first metatarsal head. Two inertial sensors (Xsens, Acc + Gyro) placed on anterior side of the proximal and distal leg. sEMG electrodes placed on residual muscles of the amputated leg. Each patient is asked to walk.	IMUs demonstrated reliable in detecting onset of gait in both healthy (Gyro) and prosthetic limb (Acc).
Simonetti E. et al., 2021 [38]	Case Study	1 TFA	To use a framework of MIMUs to evaluate BCoM acceleration and instantaneous velocity. Validate the measure versus a 3D MDS.	Full body marker set + 7 MIMUs on feet, shanks, thighs and trunk. The participant is requested to walk at self-selected speed through an 8 m path with 3 force platform in the middle.	Moderate to Strong correlation between MIMUs and Force Platform for SCoMs and BCoM acceleration and velocity.
Paradisi F. et al., 2019 [39]	Case-Control Study	20 TTA,20 Healthy Control	To investigate upper body acceleration and how these propagate from pelvis to head.	3 MIMUs located at head, sternum, and lumbo-pelvic segment. Participants were asked to walk thorough a 10 m pathway at self-selected speed.	Amputees have a larger coefficient of attenuation of acceleration from pelvis to sternum, greater medio-lateral and head acceleration. Attenuation coefficient may be a useful index for mobility assessment in LLA.
Dauriac B. et al., 2019 [40]	Case study	9 TFA	To evaluate the walking speed by estimating COM speed during gait cycle using a single IMU integrated in a microprocessor-controlled knee ankle prosthesis.	Several sped and slop conditions were tested at treadmill	This method estimates the walking speed with a 9% of RMSE in patients walking on a treadmill with 0° slope. The RMSE slightly increased when the slope is taken to 5% (but still acceptable).
Major M.J. et al., 2016 [41]	Controlled Trial	20 LLP (8 TFA, 9 TTA, 2 TT/TTA, 1 TT/TFA), 5 Healthy Control	To asses step length (SL) in patients with LLP.	A three-axis accelerometer was fixed at lumbar level in 20 LLP. The patients were asked to walk in a 20-m pathway from a standing position to a complete stop.	SL was correlated positively with previous literature study. Method was validated only on Healthy subject but not for LLP users.
Howcroft J. et al., 2014 [42]	Case study	11 TTA	To investigate if accelerometer derivate measures can differentiate between dynamic states and how those data correlate with clinical measures scores.	Community Balance and Mobility scales, Balance Berg scales, Prosthesis Evaluation Questionnaire were administered to the participants. An inertial sensor was affixed to the pelvis and then the participants walked in two scenarios: a 10-metre path on level ground (LG) and an 8-metre path covered by foam mattresses (uneven ground—UG).	Statistically significant differences were found between LG and UG walking in TTA participants. Stride time, vertical and AP acceleration FFT first quartile and ML Harmonic ratio were greater in UG than LG. Vertical acceleration and cadence were greater in LG than UG. ML acceleration range, AP acceleration standard deviation and stride time were correlated with change in clinical outcome measures scales.
Lamoth C.J. et al., 2010 [43]	Controlled Trial	8 TFA, 8 Healthy Control	To asses variability and stability of gait in LLA patients and healthy subjects.	All participants were equipped with a tri-axial accelerometer and walked for 6 min in various context: (i) indoor walking, (ii) indoor walking with cognitive dual tasks, (iii) outside walking (even terrain) in a square circuit (260 m long), (iv) outside walking (uneven terrain) in a square circuit (260 m long).	There was significant statistically differences in trunk acceleration (variability on ML acceleration) and walking speed (LLA patients are slower than healthy subject) in amputees’ group. Those two parameters are directly correlated with stability of the gait.
Tura A. et al., 2010 [44]	Controlled Trial	10 TFA, 10 Healthy Control	To evaluate a method for assessing gait regularity and symmetry of LLP users using a single accelerometer.	All participants were equipped with a single tri-axial accelerometer mounted at thoracic level and foot insoles. Patients are asked to walk a straight path 70 m long at natural, lower and faster speed. Step and stride regularity and duration were used to determine symmetry and regularity of the gait.	Step and stride regularity and step and stride duration are good index of regularity and symmetry of gait. A single accelerometer is capable to determine these parameters with good sensibility and specificity.
Clemens S. et al., 2020 [45]	Cohort Study	65 TTA, 63 TFA	To evaluate test-retest reliability of IMU based measures of segmental symmetry between lower limbs and differences between TTA and TFA in segmental symmetry score (SSS) and segmental repeatability score (SRS).	Participants wore knee sleeves equipped with 4 IMUs. They were asked to undergo a 10MWT on a Zeno Electronic Walkway system. Using sagittal angular velocities of thigh and shank SSS and SRS where calculated.	Good test-retest reliability, can differentiate between healthy and AL. Cannot differentiate between TFA and TTA.
Daines K.J.F. et al., 2021 [46]	Cohort Study	89 LLA (4 BiTTA, 1 TT/TFA, 63 TTA, 18 TFA, 2 TKA, 1 TAA)	To evaluate if the use of a random forest classificator is able to classify risk of fall in LLA.	An android smartphone was placed in posterior pelvis. All patients performed a 6MWT in a 20 m pathway. Data were collected in a custom-made application installed on to the smartphone.	Random forest classificator applied to data collected with a smartphone showed a good specificity (near 95%), good accuracy (81.3%) in classifying risk of fall in LLA patients.
Shawen N. et al., 2017 [47]	Controlled Trial	7 TFA, 10 Healthy Controls	To develop a classifier that integrates data from healthy participants to detect falls in individual with LLA.	All participants carried a Galaxy S4 Smartphone (Acc + Gyro) in natural position (pocket, hand, waist) during activities of daily living. 3 LLA participants took the phone with them for three days for quantifying false alarms.	Using a machine learning approach, data recorded from smartphones regarding angular and linear accelerations of healthy subjects can be used to classify falls risk in LLA subjects more specifically than a threshold approach (2 false alarms vs. 122).
Hordacre B. et al., 2015 [48]	Cohort Study	47 TFA	To asses activity and participation at home and various settings both for fallers and non- fallers LLA.	All participants were equipped with a stepwatch 3 activity monitor sensor and a GPS linked to the prosthesis. The community activity was defined as counting steps outside the house in various settings, and the home activity as counting steps inside the house. Participation was defined as an event in which participants had to leave home.	A statistically significant difference was demonstrated between LLA fallers versus non-fallers participants for commercial activity, recreational activity and total community activity. In addition, a statistically significant difference was found in recreational and total community participation.
Kapti et al., 2013 [49]	Case study	1 Healthy Subject	To investigate the use of accelerometric data recorded from TTA for trajectory control of an experimental active ankle joint prosthesis	Two acceleration sensors were used to register AP, ML and Vertical acceleration on the sound leg.	Data acquired from registration from the sound leg of a TTA may be used for controlling the trajectory of an LLP with active ankle joint users.
Chang M. et al., 2019 [50]	Case Study	4 TTA	To use a fuzzy logic system for terrain detection and automatic prosthetic ankle angle correction.	All participants wore a prosthesis with smart ankle system (equipped with an IMU sensor and a load cell for GRF detection) and walked on five different terrain condition (flat, upslope, downslope, upstairs and downstair) for at least 20 steps.	This fuzzy logic system had a 97.5% accuracy in terrain detection.
Su B.Y. et al., 2019 [51]	Case Study	1 TFA, 10 Healthy Participants	To evaluate a new method for training and intent recognition system using Convolutional Neural Network (CNN) algorithm.	Three IMUs were positioned on thigh, shank and ankle of the healthy leg. All participants were instructed to walk at a comfortable speed and walked among different motion states as well as steady state.	CNN can be used effectively for intent recognition with a system of 3 IMUs, and potentially to control a powered prosthesis for allowing natural transition trough motion states.
Keri MI et al., 2021 [52]	Case Study	1 TFA	To develop a low cost IMU based vibratory feedback system and use it to trigger prosthesis motion illusion (kinesthetics illusion -KI).	Vibratory feedback system (VFS) was composed with: an Arduino microcontroller, two 3-DOF Gyroscope, a lithium battery, a vibratory actuator. The accuracy of the VFS is quantified using an MDS and commercial IMU. Vibratory actuator was fixed on thigh and IMUs to a Robotic arm.	Participant in this study experienced KI for 16 degrees in knee flexion. Illusion of motion may improve gait parameters and reduce risk of falling.
Krasoulis A. et al., 2019 [53]	Controlled Trial	12 Healthy controls, 2 TRA	To develop a multi-grip classification system for prosthesis control in TRP users.	For HC 16 EMG-IMU sensors were placed in two 8 Sensor row on the forearm. For TRA 12 and 13 sensor were placed on the stump. Participants were asked to execute different grasp for calibration (power grasp, lateral grasp, tripod grasp, index pointer and hand opening). Consequentially they were asked to pick an object that stimulate a specific grasp.	Authors developed a multi-grip classification system using only two EMG-IMU sensors that can be used for real time prosthesis control during grip tasks.
Sharba G.K. et al., 2019 [54]	Controlled Trial	4 Healthy Control, 1 HlULA	To develop a RT shoulder girdle movement classifier to help high level ULA to control a prosthetic hand.	EMG and 3DOF Acc. were fixed on shoulder girdle of all participants. A set of five motions were chosen for classification: (i) elevation, (ii) depression, (iii) protraction, (iv) retraction and rest. The above classification was the used to control elbow, wrist and fingers of a 3D printed prosthesis.	Results showed a 92.8% accuracy in classifying shoulder girdle movement of the ULA participants. Classification was used to control a 3D printed UL prosthesis.
Ladlow P. et al., 2019 [55]	Controlled Trial	19 LLA (9 ULLA and 10 BiLLA), 9 Healthy Control	To asses validity of an algorithm combining data from accelerometer and HR (GT3X+ + Polar T31) monitor to assess energy expenditure (EE) during Physical Activity versus Actiheart Monitor (AHR)	All participants wore a Metamax 3B mask for Indirect calorimetry and were equipped with an AHR and a Polar T31 HR monitor. An Actigraph GTX3+ (3-DOF Acc) mounted on the waist near the shortest residual limb. All participants, then, are asked to walk on a treadmill at 5 progressive velocities and two slope (2% and 5%). Physiological Cost Index is then calculated (ΔHR/Walking speed).	The use of integrated Acc. data and HR data provided the most valid estimation of EE in ambulatorial setting for both amputation group. Level amputation impacts on accuracy of predicting EE.
Ladlow P. et al., 2017 [56]	Controlled trial	10 ULLA,10 BiLLA, 10 Healthy Control	To assess the impact of anatomical positioning of GT3X+ activity monitor in LLA participants and to develop algorithm on predicting EE.	All participants wore a Metamax 3B mask for Indirect calorimetry and were equipped with a GT3X+ activity monitor on either side of the waist above the hip and at L2 level. Participants were asked to walk on a treadmill at 5 progressive speeds. Moreover, all participants performed a sitting-based arm crank ergometry.	The anatomical positioning of accelerometers impacts the ability to predict EE in LLA. The positioning that better correlates with EE is on the amputated side of the waist, just above the hip.
Smith J.D. et al., 2021 [57]	Cross-sectional Study	23 TTA 9 TFA 3 BiTTA	To determine step count and step count accuracy with different activity monitor and O2 consumption during a 2MWT.	All participants were equipped with an Actigraph GT9X+ and a Garmin Vivofit ^®^ 3 both on wrist and ankle of the non-dominant side. A modus Stepwatch 4 is placed on the non-dominant ankle in addition to the above sensors. All Participants are fitted with a Polar HR sensor and a Cosmed 5 portable metabolic analyzer. After three minutes sitting, participants performed a 2MWT as fast and safetly possible.	There were no differences in distance walked, VO2, HR and RPE between different amputation level. Step count and cadence were greater in TTA vs. TFA. Stepwatch on the ankle and Vivofit on the wrist provided the most accurate step count.
Desveaux L. et al., 2016 [58]	Cross-sectional	15 TTA	To asses if TTA patients with diabetes meet recommended level of physical activity and daily steps count. To investigate if physical functioning measures are correlated with objective measures of physical activity.	Participants were provided with a Stepwatch activity monitor (SAM) fixed around the ankle of intact limb. Participants were asked to wear SAM for 9 consecutive days. Physical activity was measured by steps count and number of minutes engaging activity involving >90 steps/min. Each participants underwent a 2MWT and performed an L test. Activities-specific Balance Confidence Scale (ABC) and WHO QoL-Brief Questionnaire were administered.	Despite improvement in functional mobility (L test) over 6-month follow-up, step count were below 6500/day and participants spent <150 min/week for vigorous physical activity (>90 steps/min). These results indicate the needs of post-rehabilitation intervention to promote active lifestyles.
Kim J. et al., 2021 [59]	Randomized Cross-over Trial	10 TTA	To quantifying metabolic cost, step count, walking, perception of mobility and quality of life between powered and non-powered prostheses users.	Participants were randomly assigned to perform testing with a powered prosthesis or with an unpowered prosthesis. All participants were equipped with two ActiGraph GT9X Link (one mounted on the prosthetic foot and one mounted on the prosthetic pylon) and a GPS enabled system on their phone active for two weeks. At the end of the two weeks, data were collected and participants underwent a metabolic measurement with Kosmed K4b2.	Authors did not find any differences in metabolic cost between powered prosthesis.

**Table 2:** Methodological assessment of the included studies.

Articles	Criteria for the Quality Scoring										Score
	1	2	3	4	5	6	7	8	9	10	
Beausoleil S. et al., 2019 [34]	1	1	1	1	1	1	1	1	1	1	**10**
Maqbool H.F. et al., 2017 [35]	1	1	0	1	1	1	0	1	1	0	**7**
Seel T. et al., 2014 [36]	1	0	0	1	1	1	0	0	1	0	**5**
Wentink E.C. et al., 2014 [37]	1	1	1	1	1	1	1	1	1	1	**10**
Simonetti E. et al., 2021 [38]	1	1	0	1	1	1	1	1	1	1	**9**
Paradisi F. et al., 2019 [39]	1	1	0	1	1	1	1	1	1	1	**9**
Dauriac B. et al., 2019 [40]	1	1	0	1	1	1	1	1	1	1	**9**
Major M.J. et al., 2016 [41]	1	1	0	1	1	1	1	1	1	1	**9**
Howcroft J. et al., 2014 [42]	1	1	0	1	1	1	1	0	1	0	**7**
Lamoth C.J. et al., 2010 [43]	1	1	0	1	0	1	1	1	1	0	**7**
Tura A. et al., 2010 [44]	1	1	0	1	0	1	1	1	1	0	**7**
Clemens S. et al., 2020 [45]	1	1	1	1	1	1	1	1	1	0	**9**
Daines K.J.F. et al., 2021 [14]	1	1	0	1	1	1	1	1	1	1	**9**
Shawen N. et al., 2017 [47]	1	1	1	1	0	1	1	1	1	1	**9**
Hordacre B. et al., 2015 [48]	1	1	1	1	1	1	1	1	1	1	**10**
Kapti A.O. et al., 2013 [49]	1	1	0	1	0	0	1	0	1	1	**6**
Chang M. et al., 2019 [50]	1	1	0	1	0	1	0	0	1	0	**5**
Su B.Y. et al., 2019 [51]	1	1	0	1	0	1	1	1	1	0	**7**
Keri MI et al., 2021 [52]	1	1	0	1	0	1	1	1	1	1	**8**
Krasoulis A. et al., 2019 [53]	1	1	0	1	0	1	1	1	1	0	**7**
Sharba G.K. et al., 2019 [54]	1	1	0	1	0	0	1	1	1	1	**7**
Ladlow P. et al., 2019 [55]	1	1	1	1	1	1	1	1	1	1	**10**
Ladlow P. et al., 2017 [56]	1	1	1	1	1	1	1	1	1	1	**10**
Smith J.D. et al., 2021 [57]	1	1	1	1	1	1	1	1	1	1	**10**
Desveaux L. et al., 2016 [58]	1	1	1	1	1	1	1	1	1	0	**9**
Kim J. et al., 2021 [59]	1	1	1	1	1	1	1	1	1	1	**10**

Abstract informative and balanced (1); presence of detailed objectives, incorporating the hypotheses of the study(2); availability of eligibility criteria (3); for the variables of interest, availability of sources of data and characteristic of measurement methods, and description of methods correspondence (when there are two or more groups) (4); quantitative variable (5); summaries characteristics of study population (6); key results focuses on study aim (7); declare limitations (8); careful interpretation of results, based on objectives, similar literature, and other relevant evidence (9); funding statement (10).

## Data Availability

Not applicable.
